# Therapeutic Interventions against Inflammatory and Angiogenic Mediators in Proliferative Diabetic Retinopathy

**DOI:** 10.1155/2012/629452

**Published:** 2012-09-17

**Authors:** Daniel Gologorsky, Aristomenis Thanos, Demetrios Vavvas

**Affiliations:** Retina Service, Department of Ophthalmology, Massachusetts Eye and Ear Infirmary, Harvard Medical School, 243 Charles Street, Boston, MA 02131, USA

## Abstract

The global prevalence of diabetes is estimated to be 336 million people, with diabetic complications contributing to significant worldwide morbidity and mortality. Diabetic retinopathy results from cumulative microvascular damage to the retina and inflammation is recognized as a critical driver of this disease process. This paper outlines the pathophysiology leading to proliferative diabetic retinopathy and highlights many of the inflammatory, angiogenic, and cytokine mediators implicated in the development and progression of this disease. We focus a detailed discussion on the current targeted therapeutic interventions used to treat diabetic retinopathy.

## 1. Introduction

The global prevalence of diabetes is estimated to be 336 million people, and this number is projected to nearly double by 2030 [1, 2]. In addition to the primary disease itself, diabetic complications are expected to have profound implications for the future of patient management. Diabetes is a disease of hyperglycemia, and diabetic retinopathy (DR) results from cumulative microvascular damage to the retina. According to the World Health Organization, DR accounts for approximately 5% of global blindness [3]. Inflammation is a critical driver of the pathophysiology of DR [4]. This paper highlights many of the inflammatory, angiogenic, and cytokine mediators implicated in the development and progression of DR and features specific and targeted therapeutic modalities to combat retinopathy.

## 2. Pathways to Damage

Two major studies, the Diabetes Control and Complications Trial (DCCT) of 1993 and the United Kingdom Prospective Diabetes Study (UKPDS) of 1998, have demonstrated that hyperglycemia is the causative etiology for DR [5, 6]. Hyperglycemia causes microvascular changes, that in turn results in retinopathy. At least four distinct biochemical pathways have been suggested for the mechanism leading to retinopathy. These include increased polyol pathway flux, increased advanced glycation end product (AGE) formation, activation of protein kinase C (PKC) isoforms, and increased hexosamine pathway flux. Taken together, these pathways result in oxidative stresses and inflammation that attenuate vascular wall integrity and result in increased vascular permeability, occlusion, and ischemia [7, 8]. These types of microvascular insults manifest in increased vascular leakage, as in nonproliferative retinopathy (NPDR), and retinal neovascularization secondary to ischemia, as in proliferative retinopathy (PDR) [9, 10].

**Figure 1:**
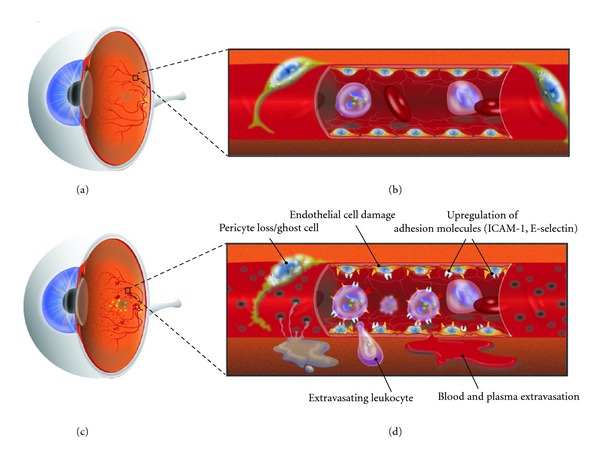
Normal eye (a) with intact vasculature (b). Accumulation of microvascular diabetic changes in the eye (c) manifest in adverse cellular changes with ultimate compromise to the blood-retinal-barrier (d).

There is increasing evidence that inflammation has a central role in the pathophysiology of diabetic retinopathy [4, 11]. Indeed, as early as 1964, it was noted that patients suffering from rheumatoid arthritis demonstrated less severe PDR if taking high dose aspirin [10]. In his review of the literature, Adamis similarly concludes that diabetic retinopathy is an inflammatory disease [4, 12]. He describes the orderly chronological progression of the disease process, briefly described here. Within a single week of experimental diabetes, prior to any clinical sign of diabetic retinopathy, infiltrating leukocytes adhere to retinal vasculature. Over time, a subset of these leukocytes accumulate and transmigrate to the retina. Using their *β*_2_ integrins, VLA-4, and CD18 surface molecules, leukocytes latch onto the local vasculature via leucocytes adhesion molecules present on the endothelium, such as intercellular adhesion molecule-1 (ICAM-1), vascular cell adhesion molecule-1 (VCAM-1), PECAM-1, and P-selectin [11–14]. In fact, early DR is marked by a disorderly upregulation of these adhesion molecules, precisely when leukocyte numbers begin to increase [15]. Once leukocytes attach to the vascular epithelium, inflammatory cytokines, growth cytokines, and vascular permeability factors are released, altering endothelial junctional proteins and allowing for leukocytic diapedesis into the retina, with concurrent compromise to the blood-retinal barrier (BRB) [4] (Figure 1).

## 3. Mediators of Damage

The upregulation of numerous factors, both angiogenic and inflammatory, has been implicated in the pathogenesis of microvascular retinopathy. Again, the expression of vascular adhesion molecules such as ICAM-1, VCAM-1, and various selectin molecules is required for leukocytic recruitment to inflammatory sites [16]. Vascular endothelial growth factor (VEGF) is an angiogenic compound that under hypoxic or ischemic conditions (as in proliferative DR) encourages aberrant vasculature. Inflammatory factors including the interleukins, tumor necrosis factor (TNF), insulin-like growth factor (IGF), angiopoietins (Ang-2), among many others have all been studied and implicated in the pathophysiological pathways leading to clinical PDR [4, 8]. These angiogenic, adhesion, and inflammatory molecules have been the focus of targeted therapies to treat DR. 

## 4. VEGF

VEGF is a member of a large family of angiogenic growth factors, a group consisting of six known members: VEGF-A (referred to as simply VEGF), placental growth factor, VEGF-B, VEGF-C, VEGF-D, and VEGF-E. VEGF-A is the first and major form involved in angiogenesis. It increases the rate of mitosis and migration of endothelial cells and is involved in integrin *α*v*β*3 regulation as well as creation of blood vessel lumen and fenestrations. In addition, it is chemotactic for macrophages and granulocytes and leads indirectly, via NO release, to vasodilation. VEGF-B is involved in embryonic angiogenesis, specifically in myocardial tissue. VEGF-C is a major prolymphangiogenesis factor, and VEGF-D is needed for the development of bronchiolar lymphatic vasculature. VEGF-E is found in viruses. PlGF is important in vasculogenesis, but plays a role in ischemia induced angiogenesis as well as inflammation and wound healing.

Though initially recognized as a vascular permeability factor, VEGF was subsequently recognized for its angiogenic properties and as a specific mitogen for vascular endothelial cells. In the context of PDR, these two findings suggest that VEGF could account for both the proliferation and vasopermeability witnessed in the disease progression. In addition to its involvement in DR, significant evidence implicates VEGF in the pathogenesis of diabetic retinopathy, retinopathy of prematurity, age-related macular degeneration, and corneal neovascularization. A simplified mechanism follows. Pathologic angiogenesis relies on the aberrant activation of proteases and various degratory enzymes emanating from the endothelium that allow for endothelial cells to leave the parental vasculature and proliferate in the matrix. Increased levels of ocular VEGF in PDR only reinforces the role of neovascularization in the course of this disease. Recent successes with anti-VEGF therapy for age-related macular degeneration in the MARINA and ANCHOR studies have prompted significant efforts to translate the application of anti-VEGF drugs to DR [17, 18].

Some forms of VEGF are more deleterious than others. Two major VEGF isoform splice variants, VEGF_120(121)_ and VEGF_164(165)_, were compared in the transparent and avascular adult mouse cornea. VEGF_164(165)_ was found to be significantly more potent at inducing corneal inflammation, stimulating ICAM-1 expression on endothelial cells, and inducing monocytic chemotaxis than VEGF_120(121)_. Of the two major VEGF isoforms, VEGF_164(165)_ was demonstrated to be more effective in inducing inflammation, neovascularization, and angiogenesis in the cornea [19]. 

As early as 1994, Aiello and Cavallerano demonstrated that individuals with PDR have elevated levels of vitreal VEGF, and that laser photocoagulation therapy significantly reduces these levels [9]. Shortly thereafter, Robinson et al. showed that blocking VEGF obviated the development of proliferative retinopathy in murine models [20]. Moreover, demonstrating the opposite effect, Tolentino et al. administered intravitreal VEGF injections and reported the ability to induce iris neovascularization and retinopathy in nonhuman primates [21].

These promising bench studies prompted efforts for a clinical intervention that would target VEGF for the treatment of PDR. Knowing that the pathophysiology of DR can be explained in the context of a leukocytic invasion with a concurrent inflammatory disorder, Lu et al. found that VEGF increases retinal vascular expression of ICAM-1 *in vivo*, and subsequent studies demonstrated that VEGF provides important chemotaxis for monocytes [22]. Joussen et al. similarly showed that retinal VEGF induces ICAM-1 expression and initiates early diabetic retinal leukocyte adhesion *in vivo*, and that blocking VEGF decreases retinal leukocyte counts in experimental diabetes [23].

VEGF has been the target of numerous drugs and clinical trials for the treatment of diabetic macular edema (DME) and PDR. VEGF inhibitors include the antibody bevacizumab (Avastin, Genentech Inc., San Francisco, USA), the monoclonal antibody fragment Ranibizumab (Lucentis, Genentech Inc., San Francisco, USA), an aptamer pegaptanib (Macugen, OSI Pharmaceuticals), the soluble VEGF receptor analogs, VEGF-Trap (Regeneron Pharmaceuticals, Tarrytown, NY, USA), small interfering RNAs (siRNAs) bevasiranib (Opko Health Inc., Miami, FL, USA), and rapamycin (Sirolimus, MacuSight Inc., Union City, CA, USA) [8]. The application of anti-VEGF medications for PDR remains off-label, as the safety and efficacy of these drugs have not been definitively established [24].

Though anti-VEGF drugs have been studied extensively for DME; no large prospective randomized studies have been published to date for the application of these agents for PDR. A retrospective study evaluating eyes with PDR treated with intravitreal bevacizumab demonstrated that complete resolution of neovascularization of the disc (NVD) was noted in 73% of the treated eyes on fluorescein angiography (FA) [25]. In 2008, Mirshahi et al. showed that 87.5% of eyes injected with bevacizumab demonstrated complete neovascularization regression at within six weeks, though this effect was temporary, as by four months the benefits of bevacizumab were strongly attenuated [26]. In 2011, Schmidinger et al. found that a 3-monthly bevacizumab retreatment regiment may be a valid method to control persistent neovascularization in PDR patients after complete panretinal photocoagulation (PRP) [27]. Other studies confirm that intravitreal bevacizumab decreases leakage from diabetic neovascular lesions and may prove to be of utility as an adjunct when it comes to vitreous hemorrhage, post-PRP macular edema, neovascularization of the iris, pars plana vitrectomy for tractional retinal detachment, nonclearing vitreous hemorrhage, and as a prevention against exacerbation of DME after cataract surgery [24, 28]. Though anti-VEGF drugs seem promising, the lack of randomized, prospective trials, standard dosing schedules, and administration protocols limits their current role to adjunctive therapies for PDR [24].

Though promising, VEGF therapy is not without risks. While numerous studies have posited the neuroprotective role of VEGF, there is a possible neurodegenerative risk with prolonged pan-VEGF blockade. Indeed, it has been shown that VEGF demonstrates neuroprotection, neurogenesis, and angiogenesis in the ischemic brain: VEGF promotes the formation of new cerebral blood vessels in response to cerebral ischemia, reduces cerebral infarct volume and edema, reduces neurologic deficits and improves neurologic recovery outcomes, and influences cerebral neurogenesis in the adult brain [29–32]. Moreover, data suggest that VEGF is endowed with anticonvulsant properties and that VEGF protects against hippocampus neuronal loss after status epilepticus [33, 34]. Though further investigation is indicated, it has been suggested that chronic pan-VEGF blockade can have deleterious effects leading to retinal neurodegeneration and choriocapillary circulatory disturbances. Indeed, VEGF inhibition or blockade may exacerbate ischemic injury and neural damage [35]. Nishijima et al. demonstrated the important role of VEGF for retinal neural survival in ischemic-reperfusion injury [36]. The same authors also noted that chronic inhibition of VEGF in normal adult animals led to a significant loss of retinal ganglion cells. These considerations must be taken into account when treating patients with VEGF for age-related macular degeneration or PDR.

## 5. ICAM 

Leukocyte adhesion to the vasculature is an important initial step in the progression of endothelial cell injury and diabetic retinopathy. This initial insult is mediated through ICAM-1 and the leukocyte integrin CD18. ICAM-1 is directly involved in immune activation and inflammation through its interaction with different cytokines, including IL-1, TNF-*α*, and IFN-*γ* [37, 38]. In 1995, McLeod et al. noted enhanced expression of ICAM-1 and P-selectin in the diabetic human retina and choroid. The authors demonstrated an increase in leucocyte density in human eyes with DR, as well as an increase in retinal vascular ICAM-1 immunoreactivity [39]. Similarly, Esser et al. demonstrated higher levels of soluble ICAM-1 in PDR and in traumatic PVR, showing concentrations that were significantly elevated above total vitreal protein levels [40]. 

Adamiec-Mroczek and Oficjalska-Młyńczak explored variations of vitreous ICAM-1, VCAM-1, IL-6, and TNF-*α* concentrations in the development of PDR [41]. The authors found that both vitreous and serum soluble adhesion molecules (ICAM-1, VCAM-1) and proinflammatory cytokine (IL-6, TNF-*α*) levels were significantly higher in patients with PDR than in controls. Further, they found that these increases in adhesion molecule levels correlated with high vitreous concentrations of IL-6 and TNF-*α* in patients with PDR, providing more evidence of the inflammatory nature of PDR. A positive correlation between vitreous soluble VCAM-1 and serum HbA1c concentrations bolstered the connection between hyperglycemia and adhesion molecule proliferation. 

While it had been previously established that increased serum levels of soluble ICAM-1, VCAM-1, and E-selectin may be found in patients with chronic inflammatory or ocular diseases, Limb et al. found that vitreous levels of ICAM-1, VCAM-1, and E-selectin were similarly significantly higher in eyes with PDR than in control cadaveric vitreous. Again, the connection between inflammation, ICAM expression, and DR is reaffirmed [42].

Barile et al. similarly measured vitreous levels of soluble ICAM-1 and VCAM-1 in the eyes of patients with retinal detachment (RD) due to proliferative diabetic retinopathy (PDR) or proliferative vitreoretinopathy (PVR). The authors found that soluble ICAM-1 and VCAM-1 are significantly increased in the vitreous cavity of patients with RD due to PDR or PVR when compared to control vitreous [43].

Research on ICAM-1 has highlighted its potential as a target of therapeutic intervention for the treatment of PDR. Joussen et al. treated animals with 50 mg/kg of aspirin, meloxicam (a cyclo-oxygenase-2 inhibitor), or etanercept (a soluble TNF-*α* receptor) [44]. The authors found that all three agents were found to reduce retinal ICAM-1 expression. Aspirin was further found to reduce the expression of CD11a, CD11b, and CD18. Each of the three agents reduced leukocyte adhesion and hindered BRB breakdown. Aspirin and meloxicam both lowered retinal TNF-*α* levels. None of the above three agents had any effect on VEGF levels. 

Recently, Hirano et al. described a novel therapeutic option for the treatment of DR by targeting ICAM-1. Hypothesizing that control over ICAM-1 expression should prevent the earliest stages of retinopathy, the authors applied small-interfering RNA (siRNAs) through a hydrodynamics-based transfection technique (HT) and intravitreal injection (IV) to a murine retina *in vivo*. Efficient modulators of gene expression, siRNAs bind to specific mammalian RNA targets and suppress target gene expression posttranscriptionally. The authors concluded that siRNA causes specific downregulation of ICAM-1 expression, suggesting a mechanism to inhibiting leukocyte infiltration and adhesion in early stage PDR [45].

Researchers have identified other promising targets related to adhesion molecules. Fasudil, a selective ROCK inhibitor, is one prime example. The Rho/ROCK pathway promotes leukocyte adhesion to the microvasculature by increasing ICAM-1 expression and affecting the function of various adhesion molecules. Intravitreal fasudil was found to reduce ICAM-1 expression, leukocyte adhesion, and endothelial apoptosis in the retinas of diabetic rats [46]. Another example is periostin, a matricellular protein with roles in cell adhesion and migration. Periostin has been associated with the formation of preretinal fibrovascular membranes, structures that form in advanced PDR that causes blindness through intravitreal hemorrhage and tractional retinal detachment. One study has suggested that targeting periostin may be a potential therapy for inhibiting fibrovascular membranes associated with PDR [47].

## 6. Inflammatory Mediators

Numerous studies have demonstrated significant increases in soluble ICAM-1 and VCAM-1 levels in patients with PDR, with corresponding elevations in vitreous IL-6 and TNF-*α* concentrations. These observations corroborate the inflammatory and immune natures of the pathophysiology of PDR. 

Two of the aforementioned studies noted an attenuation of TNF-*α* levels when treated with anti-inflammatory medications [41, 44]. This is significant because TNF-*α* plays an important role in neovascularization and vascular reactivity, in addition to its proinflammatory properties. TNF-*α* is directly involved in inflammation through an induction of cytokines, involvement in monocyte chemotaxis, and stimulation of adhesion molecules on retinal endothelium [48].

Focusing on TNF-*α*, Limb et al. measured soluble TNF-receptors (sTNF-Rs, types I and II) in patients with various retinal pathologies and found that vitreous levels of sTNF-Rs were significantly increased in eyes with PDR when compared with control eyes. Further, the authors found that the increased vitreous levels of sTNF-Rs correlate with the degree of retinopathy severity and posit that effective control of TNF-*α* activity by sTNF-Rs within the retinal microenvironment may determine the outcome and severity of retinal proliferative conditions [49].

Other studies demonstrate that inflammatory mediators cause gradual damage as retinopathy progresses. Gustavsson et al. measured levels of IL-1b, IL-6, and TNF-*α* through ELISA analysis and found that vitreous IL-6 and serum TNF-*α* levels were higher in diabetic patients than in non-diabetics. The authors concluded that intraocular inflammation is involved in PDR but does not seem to be prominent in nondiabetic retinopathy, nonproliferative diabetic retinopathy, or even in those progressing to early retinopathy stages. Those with PDR, however, had significantly more inflammatory activity, as evidenced by the increased serum levels of IL-6 and TNF-*α* [3].

Similarly, Yuuki et al. measured concentrations of IL-6, IL-8, and TNF-*α* via ELISA in the vitreous and serum of patients with PDR and vitreous noninflammatory retinopathies. Vitreous concentrations of IL-6 and IL-8 were significantly greater in patients with PDR than in noninflammatory retinopathies, and serum TNF-*α* was significantly greater in PDR than in noninflammatory retinopathies (this latter finding was limited to the serum but did not hold true in the vitreous). The authors postulated that these significant increases in IL-6, IL-8, and TNF-*α* may be diagnostically useful in PDR management [50]. Other studies have similarly found vitreal increases in IL-6 and IL-8 in PDR.

Inflammatory cytokines enhance leukocyte adhesion to endothelium, vascular permeability, and thrombus formation by inducing procoagulant and inhibiting anticoagulant, activity. Adamiec-Mroczek et al. collected vitreous and serum samples of patients with proven PDR in order to establish the role of inflammatory-proliferative processes of the endothelium in this disorder [51]. The authors found that vitreal and serum concentrations of endothelin-1 (ET-1), TNF-*α*, IL-6, vWF, and E-selectin were higher in patients with PDR than in controls. Moreover, the mean vitreous ET-1 level in the PDR patients was significantly higher than in the control group, and its serum concentration was higher in patients with PDR by a factor of seven. 

While IL-6 and IL-8 hold a prominent place in the inflammatory process, other cytokines also play prominent roles. Zhou et al. measured the vitreal concentrations of IL-1B, IL-6, IL-8, IL-10, CCL2, endothelin 1 (EDN-1), VEGF, and TNF-*α* in patients with PDR and in controls with ELISA. The authors found that with the exception of IL-10, the concentrations of all the aforementioned factors were considerably higher in PDR patients than in controls [52]. They also found a significant positive correlation between vitreous TNF-*α*, EDN1, and serum HbA1c levels in PDR patients. These results add support to the role of inflammatory cytokines and angiogenic factors in the genesis of PDR. 

Chemokines are yet another potential target for therapeutic intervention for PDR. Bian et al. demonstrated that one particular chemokine, CCL2, is an important factor in initiating leukocyte recruitment and activation, especially in the context of hyperglycemia. Levels of CCL2 are significantly elevated in the vitreous of patients with DR when compared to controls. CCL2 (also referred to as MCP-1) is the most common chemokine that is significantly elevated in the serum and vitreous [53]. Moreover, CCL-2 levels have been found to correlate with the severity and clinical stage of DR [54]. Various studies have identified other cytokines and chemokines significantly elevated in both the serum and vitreous of those suffering from DR. As CCR2 inhibitors are being studied in clinical trials to treat inflammatory disorders such as atherosclerosis, multiple sclerosis, rheumatoid arthritis, and systemic lupus erythematosus, similar chemokines are currently being studied under animal models as potential therapeutic targets for the treatment of PDR. 

While it seems that inflammatory mediators dominate the pathogenesis of PDR, other mediators and chemokines are important in the pathophysiology of the disorder. Recognizing that any growth factors present in the inert vitreous (protected by the BRB) are likely a reflection of retinal production. Pfeiffer et al. measured and found that insulin-like growth factor I (IGF-I), IGF-II, IGF binding protein 2 (IGFBP-2), and IGFBP-3 were elevated 3–13 fold in nondiabetic retinal ischemia and 1.5–3 fold in PDR [55]. Though clearly not specific to one disorder,these changes suggest that BRB breakdown and subsequent serum leakage into the vitreous is an important aspect of the pathogenesis of PDR and is a promising target for intervention.

Moreover, the same authors investigated vitreal TGF-*β*2, as it is a proposed antiangiogenic factor in the eye [55]. While the authors noted that total TGF-*β*2 levels were not altered, the active fraction of TGF-*β*2 was decreased by 30% in PDR patients. As plasmin is thought to control TGF-*β*2 activation, the authors demonstrated that serum protein *α*2-antiplasmin was significantly elevated in PDR patients to 150% of control values. This finding suggests that the flow of serum markers into the vitreous due to microvascular alterations is another potential target for therapeutic intervention. 

Microvascular changes and damage to the BRB contribute to the pathogenesis of PDR. Shiels et al. furthered the hypothesis that there is a direct relationship between plasma leakage from damaged retinal vasculature and the proliferation and phenotypic change of RPE cells with fibroblasts. These latter cells, once damaged, contribute to retinopathies by secreting matrix molecules such as fibronectin and expressing deviant surface antigens. The authors posit that control of this inflammation-induced vascular leakage would prove an important future target against microvascular damage. 

Angiopoietins, inflammatory growth factors that bind to tyrosine kinase receptors, are yet other potential targets for the treatment of BRB compromise [8]. Patel et al. attributed BRB compromise as the reason for the elevated levels of angiopoietin-2 (Ang-2) in the vitreous of patients with clinically significant macular edema (CSME) [56]. In the same vein, Rangasamy et al. found that intravitreal injection of Ang-2 in non-diabetic rats resulted in a multifold increase in retinal vascular permeability, and that Ang-2 leads to a loss of VE-cadherin function as well [57]. Fiedler et al. demonstrated that Ang-2 sensitizes endothelial cells to TNF-*α* induced expression of ICAM-1, the critical player in the pathogenesis of inflammation-induced retinopathy [58].

Proteinases are yet another class of factors involved in the progression of PDR. Parks et al. demonstrated that metalloproteinases (MMPs) are important modulators of innate immunity and inflammation, both acute and chronic [59]. Specific MMPs have been implicated in PDR. Giebel et al. found that the retinas of diabetic animals demonstrated elevated levels of MMP-2, MMP-9, and MMP-14 mRNA, and that the production of MMP-9 was especially increased in cells exposed to a hyperglycemic environment. Ultimately, cells treated with purified MMP-2 or MMP-9 demonstrated degradation of occludin, a tight junction protein [60]. Jin et al. also found that vitreal levels of MMP-9 were higher in diabetic patients with retinopathy than in controls [61]. Navaratna et al. found that the proteolytic degradation of VE-cadherin, a cell-to-cell junction protein, alters the blood-retinal barrier in diabetes and decreases vascular permeability [62]. The ramification of these studies suggests that MMPs are an important potential target for the control of PDR progression. 

Many other factors related to inflammation play a role in PDR. Augustin et al. found that lipid peroxide levels and myeloperoxidase activity was elevated in patients with PDR, suggesting the role of oxygen free radicals complementing the inflammatory pathogenesis of diabetic retinopathy [63].

## 7. Other Targets

Another target to prevent retinal angiogenesis and neovascularization in the context of PDR has been aimed at protein kinase C (PKC). The PKC enzymes, especially the beta isoforms, are found in high levels in the retina. Activator molecules, often induced by tissue hypoxia, result in increased VEGF expression. Thus, efforts have been aimed at inhibiting PKC beta enzymes, those specifically found in the retina, with low systemic toxicity. Selective inhibition of the PKC beta isoform prevents VEGF-mediated cell growth inutero and has been shown to reduce ischemia-related retinal neovascularization *in vivo* [64]. Indeed, Ishii et al. demonstrated that oral administration of a PKC-beta inhibitor reduces diabetes related vascular permeability and changes in retinal blood flow [65]. Various PKC inhibitor compounds have already been developed, such as ruboxistaurin, and several are in phase III clinical trials [66–72]. While the initial results of the multicenter randomized trial from the Protein Kinase C *β* Inhibitor Diabetic Retinopathy Study (PKC-DRS) group noted no statistically significant effect of ruboxistaurin at any of the three treatment doses for the progression of DR by their primary outcome measurements after a minimum followup of 3 years, they did note the effects of the PKC inhibitor on their secondary outcome, moderate visual loss (MLV), and sustained moderate visual loss (SMVL) [67]. Indeed, the PKC-DRS2 group subsequently studied this latter effect in more detail and concluded that ruboxistaurin reduces the occurrence of SMVL by 40% in patients with moderately severe to very severe nonproliferative diabetic retinopathy, while increasing the likelihood of visual improvement by a factor of two [66].

Fenofibrates and statins have recently been suggested to be a therapy for PDR, due to their secondary anti-inflammatory and oxidative properties rather than their primary effects on lipid levels. Studies have demonstrated that simvastatin treatment of diabetic rats resulted in the retinal suppression of superoxide formation and decreased expression of VEGF, angiopoietin 2, and erythropoietin [73]. Two recent major randomized clinical trials suggest the important role of fenofibrate for the treatment of PDR: the FIELD (Fenofibrate Intervention and Event Lowering in Diabetes) study and ACCORD (Action to Control Cardiovascular Risk in Diabetes)-Eye study. These trials included an aggregate of 11,388 patients with diabetes mellitus type II, of which 5,701 were treated with fenofibrate (±statin) for up to 5 years. In the FIELD study, retinopathy progression was defined as laser treatment for PDR or macular edema or an increase by ≥2 steps on the Early Treatment Diabetic Retinopathy Study (ETDRS) scale. Disease progression in the ACCORD-Eye study was defined as an increase of ≥3 steps on the ETDRS scale or proliferative disease requiring laser or vitrectomy treatment. In FIELD, fenofibrate (200 mg/day) reduced the requirements for laser therapy and was shown to arrest disease progression in patients with preexisting diabetic retinopathy. In ACCORD-Eye, fenofibrate (160 mg/day) taken with simvastatin yielded a 40% reduction in the odds of retinopathy progression when compared with simvastatin alone over 4 years. Fenofibrate reduced first laser treatment by 31% (*P* = 0.0002) and progression of diabetic retinopathy with absolute reductions of 5.0% over 5 years (*P* = 0.022, FIELD) and 3.7% over 4 years (*P* = 0.006, ACCORD-Eye) [74–77].

While the benefits of fenofibrates and statins have been discussed as they apply to PDR, the benefits of statins on those with cardiovascular disease have been previously established. In the randomized and double-blinded JUPITER trial, 17,603 men and women without diabetes or established cardiovascular disease were randomly assigned to rosuvastatin 20 mg or placebo and followed for up to 5 years as a primary endpoint. The trial demonstrated that rosuvastatin significantly reduced the incidence of major cardiovascular evidence in otherwise healthy individuals with elevated high-sensitivity C-reactive proteins [78]. Although the results suggested that rosuvastatin could also result in a small but significant risk of diabetes (as of February 2012, the USA Food and Drug Administration added a new warning to statin medications reflecting this risk), a subsequent study analyzing the data from the JUPITER trial determined that the risk of developing diabetes from statin therapy was limited to those subjects with baseline high risk of developing diabetes, including those with evidence of impaired fasting glucose, metabolic syndrome, severe obesity, or raised HbA1c. The authors emphasize that the cardiovascular and mortality benefits of statin therapy exceed the risk of diabetes in the trial population as a whole as well as in participants at increased risk of developing diabetes. Indeed, even among those with high risk of diabetes, rosuvastatin was estimated to prevent 134 heart attacks, strokes, or deaths, with an additional 54 cases of diagnosed diabetes. In the low-risk group, 86 heart attacks, strokes, or deaths were prevented, with no new cases of diabetes [79].

The role of better blood pressure control on the progression of PDR is less clear. Though the ACCORD-Eye and ADVANCE studies did not demonstrate any significant benefits of intensive blood pressure control on progression of diabetic retinopathy, the UK Prospective Diabetes Study did [75, 80–82]. 

Coumarin has also recently been investigated as a potential treatment for PDR. Mazzon et al. recognized the essential role of retinal microvascular compromise in the pathophysiology of PDR and investigated the effects of cloricromene on diabetes elicited by injection of streptozotocin in rats [83, 84]. An antiplatelet drug with vasodilatory and endothelial-preserving properties, cloricromene (ethyl 2-(8-chloro-3-(2-diethylaminoethyl)-4-methyl-2-oxochromen-7-yl)-oxyacetate), is a semisynthetic coumarin derivative that has been shown to mitigate chronic inflammation and resultant tissue damage associated with arthritis in rats [53]. The authors found that cloricromene significantly lowered retinal TNF-*α*, ICAM-1, VEGF, and nitric oxide synthase (eNOS) levels and suppressed diabetes-related BRB breakdown by 45%.

Many inhibitors have been developed to arrest the progression of glycation and advanced glycation end products (AGE). It has been shown that prolonged oxidative stresses in the context of diabetes result in the production and accumulation of AGEs, receptor-independent agents promoting vascular damage, fibrosis, and inflammation. Aminoguanidine, pyridoxamine, OPB-9195, LR-90, and alagebrium chloride are all agents developed to address AGE related damages [85, 86]. These have all demonstrated variable levels of therapeutic efficacy in diabetic complications. Most recently, Li et al. described the use of RAGE inhibitors on early diabetic retinopathy and tactile allodynia [87]. RAGE is the receptor for AGE, one of the pathway mechanisms of microvascular damage due to hyperglycemia in DR. The authors reported that RAGE fusion protein inhibited capillary degeneration, albumin accumulation in the neural retina, retinal protein nitration, tactile allodynia, diabetes-related retinal leukostasis, and ICAM-1 expression, although the effects of the latter two were not statistically significant at low doses.

This paper serves as a review of the pathophysiology leading to PDR and highlights the major interventional opportunities for the treatment of this disorder. A literature search for clinical markers associated with PDR is replete with inflammatory factors and numerous mediators currently being investigated for their roles in PDR. As unique markers associated with the pathophysiology of PDR are discovered, further studies will be needed to evaluate efficient and effective interventions targeted at deleterious mediators and pathways in order to obviate microvascular disease from progressing to fulminant PDR. 

## References

[1] Shaw JE, Sicree RA, Zimmet PZ (2010). Global estimates of the prevalence of diabetes for 2010 and 2030. *Diabetes Research and Clinical Practice*.

[2] Whiting DR, Guariguata L, Weil C, Shaw J (2011). IDF diabetes atlas: global estimates of the prevalence of diabetes for 2011 and 2030. *Diabetes Research and Clinical Practice*.

[3] Gustavsson C, Agardh CD, Agardh E Profile of intraocular tumour necrosis factor-alpha and interleukin-6 in diabetic subjects with different degrees of diabetic retinopathy. http://www.ncbi.nlm.nih.gov/pubmed/22520269.

[4] Adamis AP (2002). Is diabetic retinopathy an inflammatory disease?. *British Journal of Ophthalmology*.

[5] Shamoon H, Duffy H, Fleischer N (1993). The effect of intensive treatment of diabetes on the development and progression of long-term complications in insulin-dependent diabetes mellitus. *The New England Journal of Medicine*.

[6] Turner R (1998). Intensive blood-glucose control with sulphonylureas or insulin compared with conventional treatment and risk of complications in patients with type 2 diabetes (UKPDS 33). *The Lancet*.

[7] Brownlee M (2005). The pathobiology of diabetic complications: a unifying mechanism. *Diabetes*.

[8] Rangasamy S, McGuire PG, Das A (2012). Diabetic retinopathy and inflammation: novel therapeutic targets. *Middle East African Journal of Ophthalmology*.

[9] Aiello LM, Cavallerano J (1994). Diabetic retinopathy. *Current therapy in Endocrinology and Metabolism*.

[10] Fong DS, Aiello L, Gardner TW (2003). Diabetic retinopathy. *Diabetes Care*.

[11] Powell EU, Field R (1964). Diabetic retinopathy and rheumatoid arthritis. *The Lancet*.

[12] Miyamoto K, Khosrof S, Bursell SE (1999). Prevention of leukostasis and vascular leakage in streptozotocin-induced diabetic retinopathy via intercellular adhesion molecule-1 inhibition. *Proceedings of the National Academy of Sciences of the United States of America*.

[13] Schroder S, Palinski W, Schmid-Schonbein GW (1991). Activated monocytes and granulocytes, capillary nonperfusion, and neovascularization in diabetic retinopathy. *The American Journal of Pathology*.

[14] Barouch FC, Miyamoto K, Allport JR (2000). Integrin-mediated neutrophil adhesion and retinal leukostasis in diabetes. *Investigative Ophthalmology and Visual Science*.

[15] Rao KMK, Hatchell DL, Cohen HJ, De la Paz MA (1997). Alterations in stimulus-induced integrin expression in peripheral blood neutrophils of patients with diabetic retinopathy. *American Journal of the Medical Sciences*.

[16] Olson JA, Whitelaw CM, McHardy KC, Pearson DWM, Forrester JV (1997). Soluble leucocyte adhesion molecules in diabetic retinopathy stimulate retinal capillary endothelial cell migration. *Diabetologia*.

[17] Rosenfeld PJ, Brown DM, Heier JS (2006). Ranibizumab for neovascular age-related macular degeneration. *The New England Journal of Medicine*.

[18] Brown DM, Kaiser PK, Michels M (2006). Ranibizumab versus verteporfin for neovascular age-related macular degeneration. *The New England Journal of Medicine*.

[19] Usui T, Ishida S, Yamashiro K (2004). VEGF164(165) as the pathological isoform: differential leukocyte and endothelial responses through VEGFR1 and VEGFR2. *Investigative Ophthalmology and Visual Science*.

[20] Robinson GS, Pierce EA, Rook SL, Foley E, Webb R, Smith LEH (1996). Oligodeoxynucleotides inhibit retinal neovascularization in a murine model of proliferative retinopathy. *Proceedings of the National Academy of Sciences of the United States of America*.

[21] Tolentino MJ, McLeod DS, Taomoto M, Otsuji T, Adamis AP, Lutty GA (2002). Pathologic features of vascular endothelial growth factor-induced retinopathy in the nonhuman primate. *American Journal of Ophthalmology*.

[22] Lu M, Perez VL, Ma N (1999). VEGF increases retinal vascular ICAM-1 expression in vivo. *Investigative Ophthalmology and Visual Science*.

[23] Joussen AM, Poulaki V, Qin W (2002). Retinal vascular endothelial growth factor induces intercellular adhesion molecule-1 and endothelial nitric oxide synthase expression and initiates early diabetic retinal leukocyte adhesion in vivo. *The American Journal of Pathology*.

[24] Waisbourd M, Goldstein M, Loewenstein A (2011). Treatment of diabetic retinopathy with anti-VEGF drugs. *Acta Ophthalmologica*.

[25] Avery RL, Pieramici DJ, Rabena MD, Castellarin AA, Nasir MA, Giust MJ (2006). Intravitreal bevacizumab (Avastin) for neovascular age-related macular degeneration. *Ophthalmology*.

[26] Mirshahi A, Roohipoor R, Lashay A, Mohammadi SF, Abdoallahi A, Faghihi H (2008). Bevacizumab-augmented retinal laser photocoagulation in proliferative diabetic retinopathy: a randomized double-masked clinical trial. *European Journal of Ophthalmology*.

[27] Schmidinger G, Maar N, Bolz M, Scholda C, Schmidt-Erfurth U (2011). Repeated intravitreal bevacizumab (AvastinÂ) treatment of persistent new vessels in proliferative diabetic retinopathy after complete panretinal photocoagulation. *Acta Ophthalmologica*.

[28] Nicholson BP, Schachat AP (2010). A review of clinical trials of anti-VEGF agents for diabetic retinopathy. *Graefe’s Archive for Clinical and Experimental Ophthalmology*.

[29] Rosenstein JM, Mani N, Silverman WF, Krum JM (1998). Patterns of brain angiogenesis after vascular endothelial growth factor administration in vitro and in vivo. *Proceedings of the National Academy of Sciences of the United States of America*.

[30] Hayashi T, Abe K, Itoyama Y (1998). Reduction of ischemic damage by application of vascular endothelial growth factor in rat brain after transient ischemia. *Journal of Cerebral Blood Flow and Metabolism*.

[31] Zhang ZG, Zhang L, Jiang Q (2000). VEGF enhances angiogenesis and promotes blood-brain barrier leakage in the ischemic brain. *Journal of Clinical Investigation*.

[32] Sun Y, Jin K, Xie L (2003). VEGF-induced neuroprotection, neurogenesis, and angiogenesis after focal cerebral ischemia. *Journal of Clinical Investigation*.

[33] Vezzani A (2008). VEGF as a target for neuroprotection. *Epilepsy Currents/American Epilepsy Society*.

[34] Nicoletti JN, Shah SK, McCloskey DP (2008). Vascular endothelial growth factor is up-regulated after status epilepticus and protects against seizure-induced neuronal loss in hippocampus. *Neuroscience*.

[35] D’Amore PA (2007). Vascular endothelial cell growth factor-A: not just for endothelial cells anymore. *American Journal of Pathology*.

[36] Nishijima K, Ng YS, Zhong L (2007). Vascular endothelial growth factor-A is a survival factor for retinal neurons and a critical neuroprotectant during the adaptive response to ischemic injury. *The American Journal of Pathology*.

[37] Adamis AP, Berman AJ (2008). Immunological mechanisms in the pathogenesis of diabetic retinopathy. *Seminars in Immunopathology*.

[38] Joussen AM, Poulaki V, Le ML (2004). A central role for inflammation in the pathogenesis of diabetic retinopathy. *The FASEB Journal*.

[39] McLeod DS, Lefer DJ, Merges C, Lutty GA (1995). Enhanced expression of intracellular adhesion molecule-1 and P-selectin in the diabetic human retina and choroid. *The American Journal of Pathology*.

[40] Esser P, Bresgen M, Fischbach R, Heimann K, Wiedemann P (1995). Intercellular adhesion molecule-1 levels in plasma and vitreous from patients with vitreoretinal disorders. *German Journal of Ophthalmology*.

[41] Adamiec-Mroczek J, Oficjalska-Młyńczak J (2008). Assessment of selected adhesion molecule and proinflammatory cytokine levels in the vitreous body of patients with type 2 diabetes—role of the inflammatory-immune process in the pathogenesis of proliferative diabetic retinopathy. *Graefe’s Archive for Clinical and Experimental Ophthalmology*.

[42] Limb GA, Hickman-Casey J, Hollifield RD, Chignell AH (1999). Vascular adhesion molecules in vitreous from eyes with proliferative diabetic retinopathy. *Investigative Ophthalmology and Visual Science*.

[43] Barile GR, Chang S, Park L, Reppucci VS, Schiff WM, Schmidt AM (1999). Soluble cellular adhesion molecules in proliferative vitreoretinopathy and proliferative diabetic retinopathy. *Current Eye Research*.

[44] Joussen AM, Poulaki V, Mitsiades N (2002). Nonsteroidal anti-inflammatory drugs prevent early diabetic retinopathy via TNF-alpha suppression. *The FASEB Journal*.

[45] Hirano Y, Sakurai E, Matsubara A, Ogura Y (2010). Suppression of ICAM-1 in retinal and choroidal endothelial cells by plasmid small-interfering RNAs in vivo. *Investigative Ophthalmology and Visual Science*.

[46] Arita R, Hata Y, Nakao S (2009). Rho kinase inhibition by fasudil ameliorates diabetes-induced microvascular damage. *Diabetes*.

[47] Yoshida S, Ishikawa K, Asato R (2011). Increased expression of periostin in vitreous and fibrovascular membranes obtained from patients with proliferative diabetic retinopathy. *Investigative Ophthalmology and Visual Science*.

[48] Elner SG, Elner VM, Bian ZM (1997). Human retinal pigment epithelial cell interleukin-8 and monocyte chemotactic protein-1 modulation by T-lymphocyte products. *Investigative Ophthalmology and Visual Science*.

[49] Limb GA, Hollifield RD, Webster L, Charteris DG, Chignell AH (2001). Soluble TNF receptors in vitreoretinal proliferative disease. *Investigative Ophthalmology and Visual Science*.

[50] Yuuki T, Kanda T, Kimura Y (2001). Inflammatory cytokines in vitreous fluid and serum of patients with diabetic vitreoretinopathy. *Journal of Diabetes and its Complications*.

[51] Adamiec-Mroczek J, Oficjalska-Młyńczak J, Misiuk-Hojło M (2009). Proliferative diabetic retinopathy-The influence of diabetes control on the activation of the intraocular molecule system. *Diabetes Research and Clinical Practice*.

[52] Zhou J, Wang S, Xia X (2012). Role of intravitreal inflammatory cytokines and angiogenic factors in proliferative diabetic retinopathy. *Current eye Research*.

[53] Capeans C, De Rojas MV, Lojo S, Salorio MS (1998). C-C chemokines in the vitreous of patients with proliferative vitreoretinopathy and proliferative diabetic retinopathy. *Retina*.

[54] Tashimo A, Mitamura Y, Nagai S (2004). Aqueous levels of macrophage migration inhibitory factor and monocyte chemotactic protein-1 in patients with diabetic retinopathy. *Diabetic Medicine*.

[55] Pfeiffer A, Spranger J, Meyer-Schwickerath R, Schatz H (1997). Growth factor alterations in advanced diabetic retinopathy: a possible role of blood retina barrier breakdown. *Diabetes*.

[56] Patel JI, Hykin PG, Gregor ZJ, Boulton M, Cree IA (2005). Angiopoietin concentrations in diabetic retinopathy. *British Journal of Ophthalmology*.

[57] Rangasamy S, Srinivasan R, Maestas J, McGuire PG, Das A A potential role for angiopoietin 2 in the regulation of the blood-retinal barrier in diabetic retinopathy. *Investigative Ophthalmology and Visual Science*.

[58] Fiedler U, Reiss Y, Scharpfenecker M (2006). Angiopoietin-2 sensitizes endothelial cells to TNF-*α* and has a crucial role in the induction of inflammation. *Nature Medicine*.

[59] Parks WC, Wilson CL, López-Boado YS (2004). Matrix metalloproteinases as modulators of inflammation and innate immunity. *Nature Reviews Immunology*.

[60] Giebel SJ, Menicucci G, McGuire PG, Das A (2005). Matrix metalloproteinases in early diabetic retinopathy and their role in alternation of the blood-retinal barrier. *Laboratory Investigation*.

[61] Jin M, Kashiwagi K, Iizuka Y, Tanaka Y, Imai M, Tsukahara S (2001). Matrix metalloproteinases in human diabetic and nondiabetic vitreous. *Retina*.

[62] Navaratna D, McGuire PG, Menicucci G, Das A (2007). Proteolytic degradation of VE-cadherin alters the blood-retinal barrier in diabetes. *Diabetes*.

[63] Augustin AJ, Breipohl W, Boker T, Lutz J, Spitznas M (1993). Increased lipid peroxide levels and myeloperoxidase activity in the vitreous of patients suffering from proliferative diabetic retinopathy. *Graefe’s Archive for Clinical and Experimental Ophthalmology*.

[64] Geraldes P, Hiraoka-Yamamoto J, Matsumoto M (2009). Activation of PKC-and SHP-1 by hyperglycemia causes vascular cell apoptosis and diabetic retinopathy. *Nature Medicine*.

[65] Ishii H, Jirousek MR, Koya D (1996). Amelioration of vascular dysfunctions in diabetic rats by an oral PKC *β* inhibitor. *Science*.

[66] Aiello LP (2005). The effect of ruboxistaurin on visual loss in patients with moderately severe to very severe nonproliferative diabetic retinopathy: initial results of the protein kinase C *β* inhibitor diabetic retinopathy study (PKC-DRS) multicenter randomized clinical trial. *Diabetes*.

[67] Aiello LP, Davis MD, Girach A (2006). Effect of ruboxistaurin on visual loss in patients with diabetic retinopathy. *Ophthalmology*.

[68] Titchenell PM, Lin CM, Keil JM, Sundstrom JM, Smith CD, Antonetti DA (2012). Novel atypical PKC inhibitors prevent vascular endothelial growth factor-induced blood-retinal barrier dysfunction. *The Biochemical Journal*.

[69] Pathak D, Gupta A, Kamble B, Kuppusamy G, Suresh B (2012). Oral targeting of protein kinase C receptor: promising route for diabetic retinopathy?. *Current Drug Delivery*.

[70] Frank RN (2002). Potential new medical therapies for diabetic retinopathy: protein kinase C inhibitors. *American Journal of Ophthalmology*.

[71] Gálvez MIL (2009). Rubosixtaurin and other pkc inhibitors in diabetic retinopathy and macular Edema. Review. *Current Diabetes Reviews*.

[72] Aiello LP (2002). The potential role of PKC *β* in diabetic retinopathy and macular edema. *Survey of Ophthalmology*.

[73] Lee SG, Kim JL, Lee HK (2011). Simvastatin suppresses expression of angiogenic factors in the retinas of rats with streptozotocin-induced diabetes. *Graefe’s Archive for Clinical and Experimental Ophthalmology*.

[74] Keech A, Mitchell P, Summanen P (2007). Effect of fenofibrate on the need for laser treatment for diabetic retinopathy (FIELD study): a randomised controlled trial. *The Lancet*.

[75] Chew EY, Ambrosius WT, Davis MD (2010). Effects of medical therapies on retinopathy progression in type 2 diabetes. *The New England Journal of Medicine*.

[76] Wong TY, Simo R, Mitchell P (2012). Fenofibrate—a potential systemic treatment for diabetic retinopathy?. *American Journal of Ophthalmology*.

[77] Wright AD, Dodson PM (2011). Medical management of diabetic retinopathy: fenofibrate and ACCORD Eye studies. *Eye*.

[78] Ridker PM, Danielson E, Fonseca FAH (2008). Rosuvastatin to prevent vascular events in men and women with elevated C-reactive protein. *The New England Journal of Medicine*.

[79] Ridker PM, Pradhan A, MacFadyen JG (2012). Cardiovascular benefits and diabetes risks of statin therapy in primary prevention: an analysis from the JUPITER trial. *The Lancet*.

[80] UK Prospective Diabetes Study Group (1999). Tight blood pressure control and risk of macrovascular and microvascular complications in type 2 diabetes: UKPDS 38. *British Medical Journal*.

[81] Patel A, MacMahon S, Chalmers J (2008). Intensive blood glucose control and vascular outcomes in patients with type 2 diabetes. *The New England Journal of Medicine*.

[82] Beulens JWJ, Patel A, Vingerling JR (2009). Effects of blood pressure lowering and intensive glucose control on the incidence and progression of retinopathy in patients with type 2 diabetes mellitus: a randomised controlled trial. *Diabetologia*.

[83] Bucolo C, Ward KW, Mazzon E, Cuzzocrea S, Drago F (2009). Protective effects of a coumarin derivative in diabetic rats. *Investigative Ophthalmology and Visual Science*.

[84] Cuzzocrea S, Mazzon E, Bevilaqua C (2000). Cloricromene, a coumarine derivative, protects against collagen-induced arthritis in Lewis rats. *British Journal of Pharmacology*.

[85] Thomas MC, Baynes JW, Thorpe SR, Cooper ME (2005). The role of AGEs and AGE inhibitors in diabetic cardiovascular disease. *Current Drug Targets*.

[86] Zong H, Ward M, Stitt AW (2011). AGEs, RAGE, and diabetic retinopathy. *Current Diabetes Reports*.

[87] Li G, Tang J, Du Y, Lee CA, Kern TS (2011). Beneficial effects of a novel RAGE inhibitor on early diabetic retinopathy and tactile allodynia. *Molecular Vision*.

